# Neuroanatomical Heterogeneity of Essential Tremor According to Propranolol Response

**DOI:** 10.1371/journal.pone.0084054

**Published:** 2013-12-16

**Authors:** Seok Jong Chung, Hunki Kwon, Dong-Kyun Lee, Jin Yong Hong, Mun-Kyung Sunwoo, Young H. Sohn, Jong-Min Lee, Phil Hyu Lee

**Affiliations:** 1 Department of Neurology, Yonsei University College of Medicine, Seoul, Korea; 2 Department of Biomedical Engineering, Hanyang University, Seoul, Korea; 3 Department of Neurology, Yonsei University Wonju College of Medicine, Wonju, Korea; Hospital General Dr. Manuel Gea González, Mexico

## Abstract

**Background:**

Recent studies have suggested that essential tremor (ET) is a more complex and heterogeneous clinical entity than initially thought. In the present study, we assessed the pattern of cortical thickness and diffusion tensor white matter (WM) changes in patients with ET according to the response to propranolol to explore the pathogenesis underlying the clinical heterogeneity of ET.

**Methods:**

A total of 32 patients with drug naive ET were recruited prospectively from the Movement Disorders outpatient clinic. The patients were divided into a propranolol-responder group (*n* = 18) and a non-responder group (*n* = 14). We analyzed the pattern of cortical thickness and diffusion tensor WM changes between these two groups and performed correlation analysis between imaging and clinical parameters.

**Results:**

There were no significant differences in demographic characteristics, general cognition, or results of detailed neuropsychological tests between the groups. The non-responder group showed more severe cortical atrophy in the left orbitofrontal cortex and right temporal cortex relative to responders. However, the responders exhibited significantly lower fractional anisotropy values in the bilateral frontal, corpus callosal, and right parietotemporal WM compared with the non-responder group. There were no significant clusters where the cortical thickness or WM alterations were significantly correlated with initial tremor severity or disease duration.

**Conclusions:**

The present data suggest that patients with ET have heterogeneous cortical thinning and WM alteration with respect to responsiveness to propranolol, suggesting that propranolol responsiveness may be a predictive factor to determine ET subtypes in terms of neuroanatomical heterogeneity.

## Introduction

Essential tremor (ET) is one of the most common movement disorders and has been widely regarded as a monosymptomatic condition, characterized by kinetic arm tremor [1], [2]. However, as our understanding of ET is advancing, the concept of ET as a more complex and heterogeneous clinical entity has been rapidly gaining acceptance, although evidence suggesting that ET is likely a neurodegenerative disorder is still inconclusive. Several clinical series indicated that patients with ET had additional neurological signs including cognitive impairment, cerebellar disturbances, and olfactory deficits [3]–[6]. Additionally, the neuropathological studies indicated that the majority of ET cases have changes in the cerebellum, whereas some had Lewy bodies (LB) or neuronal depletion in the brainstem, mainly in the locus coeruleus (LC) [7]–[9].

The pathophysiology of ET also remains unclear. One possible explanation is that the abnormal intrinsic oscillations influence the cerebello–thalamo–cortical loop. Several neuroimaging studies using positron emission tomography (PET), voxel-based morphometry, and diffusion tensor imaging (DTI) in patients with ET supported the disintegration of this loop [10]–[18]. However, no neuroimaging studies have evaluated the neuroanatomical substrates for complex clinical characteristics of ET. Only recently have postmortem studies attempted to correlate the heterogeneous clinicopathological findings, and these showed that some clinical characteristics tended to differ between ET patients with and without LB pathologies [7], [8].

We hypothesized that the variable responsiveness to propranolol, which is one of the most widely used and efficacious anti-tremor drugs, may reflect underlying structural or functional changes in individuals with ET. Indeed, about 30% of patients with ET do not respond to propranolol, and some cases show tolerance to the drug effect with chronic treatment [19]. Therefore, we assessed the pattern of cortical thickness and diffusion tensor white matter (WM) changes in patients with ET according to the response to propranolol to explore the pathogenesis underlying the clinical heterogeneity of ET.

## Patients and Methods

### Subjects

The study population consisted of 32 patients with drug naive ET recruited from the Movement Disorders outpatient clinic at Severance Hospital, Seoul, Korea, between March and October 2012. ET was diagnosed according to the criteria of the Movement Disorder Society on Tremor [20]. Patients had no history of exposure to ET medications, such as beta-blockers and primidone. Each subject underwent brain magnetic resonance imaging (MRI), the cross-cultural smell identification test (CCSIT) [21], and a neuropsychological test battery. The severity of ET was assessed with the Fahn–Tolosa–Marin tremor rating scale (TRS) [22].

Exclusion criteria included the presence of medical comorbidities interfering with the use of beta-blockers (e.g., asthma, atrioventricular block), other neurological signs (e.g., dystonia, parkinsonian features), a history of exposure to tremorgenic drugs (e.g., gastrointestinal drugs, neuroleptics), and evidence of focal brain lesions, multiple lacunes, or diffuse areas of WM hyperintensity on brain MRI.

This study was approved by the Yonsei University Severance Hospital institutional review board. Written informed consent was obtained from all subjects who participated in this study.

### Assessment of response to propranolol

Each subject was initially treated with the beta-blocker, propranolol, with escalation of dose from 40 mg/day to 80 mg/day after 2 weeks, which was widely established as a standard care for ET. Movement disorders specialists (Y.H.S. and P.H.L. who involved in the current study) prescribed propranolol in the outpatient clinic. The severity of tremor was assessed twice with the Fahn–Tolosa–Marin TRS for all patients, i.e., at baseline on the first visit and on the second visit after 8 weeks of medication. The Fahn–Tolosa–Marin TRS consisted of three parts: Part A (amplitude of tremor in different body parts); Part B (tremor in writing, drawing, and pouring); and Part C (functional disabilities in daily living). Each item was scored from 0 (none) to 4 (severe), and the total possible score was 156. The improvement of total TRS scores after 8 weeks (response rate) was calculated. The patients were then divided into two groups according to the response to propranolol: responders, over 25% improvement in total TRS scores; non-responders, below 25% improvement in total TRS scores [23].

### Neuropsychological assessment

All subjects were administered the Seoul Neuropsychological Screening Battery (SNSB) [24], which is a detailed Korean language neuropsychological test battery consisting of five cognitive domains: attention (forward/backward digit span and letter cancellation); language and related functions (Korean version of the Boston Naming Test [K-BNT], calculation, and praxis); visuospatial function (Rey Complex Figure Test [RCFT]); memory (three-word registration/recall and Seoul Verbal Learning Test [SVLT] for verbal memory; immediate recall/delayed recall/recognition using RCFT for visual memory); and frontal/executive function (contrasting program, go/no-go test, Controlled Oral Word Association Test [COWAT], and Stroop test). The scores for each cognitive domain were determined as abnormal when they were below the 16^th^ percentile of the age-, sex-, and education-specific norms of 447 healthy control subjects.

### MRI acquisition

All MRI scans of ET patients were acquired using a Philips 3.0 T scanner (Philips Intera; Philips Medical System, Best, The Netherlands) with a SENSE head coil (SENSE factor  = 2). The high-resolution T1-weighted MRI data were obtained axially from all subjects using a 3D T1-TFE sequence with the following parameters: 224×224 axial acquisition matrix; 256×256 reconstructed matrix with 170 slices; field of view, 220 mm; voxel size, 0.859×0.859×1 mm^3^; echo time, 4.6 ms; repetition time, 9.8 ms; flip angle, 8°. The diffusion-weighted MRI data were obtained from 30 subjects using a single-shot echo-planar acquisition with the following parameters: 45 non-collinear, non-coplanar diffusion-encoded gradient directions; 128×128 acquisition matrix with 70 slices; field of view, 220 mm; voxel size, 1.75×1.75×2 mm^3^; echo time, 70 ms; repetition time, 7.663 s; flip angle, 90°; b-factor, 600 s/mm^2^.

### Analysis of cortical thickness

The following steps were applied to high-resolution T1-weighted MRI data, which have been described in detail elsewhere [25]–[29]. A fully 3D technique for inhomogeneity correction removed a serious obstacle for automated segmentation of MRI, which slowly varied the change in signal intensity over the image caused by magnetic field inhomogeneity [26]. To account for interindividual differences in absolute brain size, each brain was separately transformed to a standardized stereotactic space and resampled on a 1-mm^3^ voxel grid. This procedure was performed with automatic registration software using a 3D cross-correlation approach to match the single MRI volume, with the intensity average of 305 MRI brain volumes previously aligned in a standardized stereotactic space [25]. An artificial neural network classifier was applied to identify gray matter (GM), WM, and cerebrospinal fluid (CSF) [28]. The cortical surface was automatically extracted from each MR volume using the Constrained Laplacian-based Automated Segmentation with Proximities (CLASP) algorithm. Cortical thickness was calculated as the Euclidean distance between the corresponding vertices of the inner and outer cortical surfaces [30].

We analyzed the global difference and corrected *t*-statistical maps of cortical thickness between the groups, adjusting for age, sex, years of education, disease duration, and intracranial volume as covariates. Statistical analyses were performed using SurfStat toolbox (http://www.math.mcgill.ca/keith/surfstat/), for Matlab (R2008b; MathWorks, Natick, MA). The results for the between-group differences in cortical thickness were considered significant at random-field theory (RFT)-corrected *P*<0.05 [31].

### DTI processing

DTI data were preprocessed using the Functional Magnetic Resonance Imaging of the Brain (FMRIB) Software Library (FSL) program (http://www.fmrib.ox.ac.uk/fsl/). Motion artifacts and eddy current distortions were corrected by normalization of each directional volume to the non-diffusion-weighted volume (b0) using the FMRIB Linear Image Registration Tool (FLIRT) with 6 degrees of freedom. After correction of motion artifacts and eddy current distortions, the diffusion tensor was calculated using a simple least-squares fit of the tensor model. Then, the fractional anisotropy (FA) and mean diffusivity (MD) were determined for each voxel using standard methods of the DTIFIT program in FSL.

### Tract-based spatial statistics (TBSS) analysis

The FA and MD maps of DTI preprocessing results were used in TBSS analysis. All FA images were aligned to the standard FMRIB58 FA template provided by the FSL program using a nonlinear registration algorithm implemented in the TBSS package. The FA images were then averaged to create a skeletonized mean FA image. Each subject's aligned FA images were projected onto this skeleton by filling the skeleton with the highest FA values from the nearest relevant center of fiber tracts [32]. A threshold FA value of 0.2 was chosen to exclude voxels of adjacent GM or CSF. The MD images were also processed using identical methods to the FA data by applying the nonlinear registration algorithm and projecting them onto the skeleton.

To compare the values for the responder and non-responder groups, voxel-wise statistical analysis of individual skeleton images was performed using a nonparametric permutation test. Age, sex, years of education, and disease duration were included as covariates in the analysis of covariance (ANCOVA). The null distribution was built up over 5000 permutations. For control over the multiple comparison correction, we used the threshold-free cluster enhancement (TFCE) approach with the 2D parameter settings [33]. The results for FA and MD were considered significant for familywise error (FWE)-corrected *P*<0.05. The FA is an index of directional selectivity of water diffusion, and the MD is the average diffusivity of three dimensions, where decreased FA and increased MD are indicative of WM disintegration.

### Correlation analysis

Multiple regression analysis was performed to determine the correlations between cortical thickness and initial tremor severity assessed by TRS or disease duration, adjusting for age, sex, years of education, and intracranial volume as covariates. The result for cortical thickness was considered significant at RFT-corrected *P*<0.05. Analysis of correlations between FA values and total TRS score or disease duration was also performed, adjusting for age, sex, and years of education. The results for FA and MD were considered significant at FWE-corrected *P*<0.05.

### Statistical analysis

To compare the baseline demographic characteristics of the two groups, the Mann–Whitney *U*-test and Fisher's exact test were used for continuous and categorical variables, respectively. Multiple linear regression analysis was used to compare the subscores of the detailed neuropsychological test, adjusting for age, sex, and years of education. Statistical analyses were performed using SPSS version 18.0 (SPSS, Inc., Chicago, IL, USA), and two-tailed *P*<0.05 was considered significant.

## Results

### Demographic characteristics

Table 1 shows the baseline demographic characteristics of the patients with ET (18 responders and 14 non-responders). There were no significant differences in age, sex, years of education, or general cognitive deficits as measured by the Korean version of the Mini-Mental State Examination (K-MMSE) [34] or Clinical Dementia Rating (CDR) [35] between the two groups. There were also no significant differences in duration of tremor, olfactory function measured by the CCSIT, or the presence of family history of ET between the two groups. Additionally, the initial tremor severity assessed by the Fahn–Tolosa–Marin TRS was not significantly different between the groups. A detailed neuropsychological test battery, the SNSB, also showed no significant differences in each cognitive domain (Table S1). Subanalyses were performed for 30 of 32 patients with ET (16 responders and 14 non-responders) for whom DTI data were available. There were no significant differences in the baseline demographic characteristics or neuropsychological test results between responders and non-responders (data not shown).

**Table 1:** Baseline demographic characteristics.

	Responder (n = 18)	Nonresponder (n = 14)	P
**Age**	62.4 (8.6)	63.2 (10.5)	0.821
**Female, No. (%)**	13 (72.2)	8 (57.1)	0.465
**Education (years)**	9.0 (0–18)	12.0 (3–18)	0.924
**Age at onset**	44.1 (16.5)	46.6 (16.1)	0.662
**Duration (years)**	15.0 (4–50)	11.5 (5–40)	0.985
**K-MMSE**	28.0 (17–30)	29.0 (19–30)	0.668
**CDR**	0.25 (0–1)	0.25 (0–1)	NS
**CDR (SOB)**	0.25 (0–3)	0.25 (0–3)	0.790
**CCSIT**	10.0 (5–12)	9.0 (4–11)	0.255
**Family history, No. (%)**	16 (88.9)	9 (64.3)	0.195
**Limb tremor, No. (%)**	18 (100)	14 (100)	NS
**Head tremor, No. (%)**	11 (61.1)	8 (57.1)	0.821
**Before medication**			
TRS A	11.5 (6–23)	12.5 (5–42)	0.819
TRS B	14.0 (2–26)	12.0 (4–36)	0.924
TRS C	7.5 (2–19)	8.0 (2–31)	0.351
TRS total	31.5 (12–67)	36.5 (12–109)	0.582
**After medication**			
TRS A	7.0 (3–17)	12.5 (5–42)	0.030
TRS B	8.0 (1–21)	12.5 (4–36)	0.031
TRS C	4.0 (0–14)	8.0 (2–31)	0.027
TRS total	21.0 (5–50)	36.5 (12–109)	0.013
**Response rate**			
TRS total	33.85 (25.0–58.7)	0.00 (−2.7–8.6)	<0.001

The values are expressed as median (minimum -maximum), mean (SD), or number (percentage). Abbreviations: K-MMSE, Korean version of Mini-Mental State Examination; CDR, Clinical Dementia Rating; SOB, Sum of Boxes; CCSIT, Cross cultural smell identification test; TRS, tremor rating scale; NS, not significant.

### Analysis of cortical thickness

**Figure 1:**
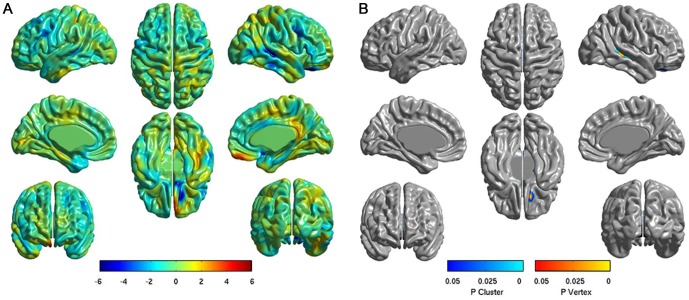
Analysis of cortical thickness in comparing responders versus non-responders. (A) Difference maps of cortical thickness between two groups. The color scale bar shows the difference in mean cortical thickness between the two groups, with blue and red indicating less and greater cortical thickness in non-responders, respectively. (B) Corrected *t*-statistical maps of cortical thickness. The non-responders had significantly less cortical thickness in the left orbital gyrus and right middle temporal gyrus compared with the responders (RFT-corrected *P*<0.05).

The mean difference maps of cortical thickness between the two groups (18 responders and 14 non-responders) are shown in Figure 1A. The color scale bar at the bottom represents the between-group differences in cortical thickness. In this color scale, blue and red indicates less and greater cortical thickness, respectively, in the non-responder group compared with the responder group. The non-responder group tended to show less cortical thickness in the left orbitofrontal cortex (OFC) and right superolateral temporal lobe. Figure 1B shows the corrected *t*-statistical maps of cortical thickness. Significant differences were observed in the left orbital gyrus and right middle temporal gyrus, where the non-responders exhibited less cortical thickness than did the responders (RFT-corrected *P*<0.05). No areas were found in which the non-responders had greater cortical thickness compared with the responders.

### TBSS analysis

**Figure 2:**
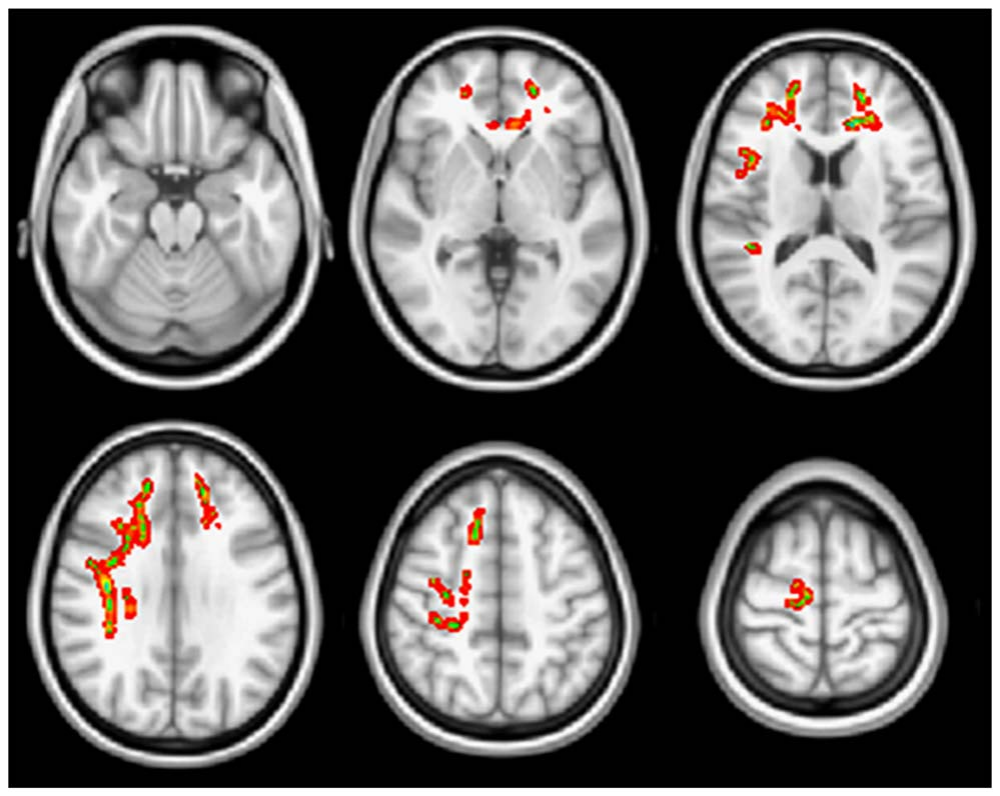
TBSS analysis of fractional anisotropy (FA) in comparison of responder and non-responder groups. Higher FA values of the non-responders compared with the responders were found in the bilateral frontal, corpus callosal (genu), and right parietotemporal WM (FWE-corrected *P*<0.05).

The non-responders exhibited significantly higher FA values in the bilateral frontal, corpus callosal (genu), and right parietotemporal WM than the responders did (FWE-corrected *P*<0.05; Figure 2). The non-responder group did not have areas with significantly reduced FA values (i.e., more severe structural WM change) compared with the responder group. There were no significant MD differences between the groups.

### Correlation analysis

No significant clusters where the cortical thickness was significantly correlated with initial tremor severity assessed by TRS were observed. Additionally, the FA values were not significantly correlated with the severity of tremor. Furthermore, the disease duration was not significantly correlated with either cortical thinning or FA value.

## Discussion

The present study demonstrated that patients with ET who had no response to propranolol exhibited reduced cortical thickness in the left orbitofrontal and middle temporal areas compared with those who responded to this agent. Our study also showed that the responders had greater disintegration of WM in the regions corresponding to the fibers of the cerebello–thalamo–cortical loop, despite the absence of significant differences in age, sex, disease duration, and cognitive level. These data suggest that patients with ET have heterogeneous cortical thinning and WM alteration with respect to responsiveness to propranolol.

Cortical thinning measurement and DTI tractography have been frequently used to investigate the patterns of anatomical connectivity in the human brain [36], [37]. These approaches have been widely applied in several neurological disorders to understand the neural correlates of each disorder [38]–[40]. Thus, heterogeneous cortical thinning and WM alteration of ET patients may reflect the different anatomical connectivity and subsequently lead to distinct clinical features.

The mechanism by which beta-blockers alleviate symptoms in patients with ET is still unclear. Some studies have suggested that beta-blockers may act on peripheral beta-adrenoreceptors in the muscle fibers or spindles [41]–[43]. Other conflicting findings supported a possible role of beta-blockers via a central action, based on the different pharmacokinetics and effects on ET of various beta-blockers [44], [45]. However, the central neuroanatomical substrate that is responsible for the pharmacological actions of beta-blockers in ET has not been established. Among several possible structures, the LC has been suggested as a strong candidate [46], [47], as it is the primary source of noradrenergic innervations and expresses beta-adrenoreceptors [48]. Recently, Baker et al. reported anti-tremor effects of beta-blockers through a central site of action [47]. Based on changes in corticomuscular coherence patterns, they speculated that beta-blockers act centrally through modulation of Renshaw cells, mediated indirectly via inputs from neurons expressing beta-adrenoreceptors, such as those in the LC. Furthermore, several series have reported a possible role of the LC in the pathogenesis of ET. Animal studies using a harmaline-induced tremor model showed that a lesion of the LC augmented the tremor [49], [50]. Recent neuropathological studies of patients with ET revealed that some cases had abundant LB or neuronal loss in the LC [7], [9]. The Purkinje cells in the cerebellum are known to be innervated by noradrenergic terminals arising from the LC with an inhibitory influence [51], [52], and this coerulo-cerebellar pathway extends projections to the subcortical cerebellar nuclei, thalamus, and cerebral cortex [53]–[55]. Therefore, any lesion in this pathway, including the LC, may lead to alteration in the function of the Purkinje cells and disintegration of the cerebello–thalamo–cortical loop.

**Figure 3:**
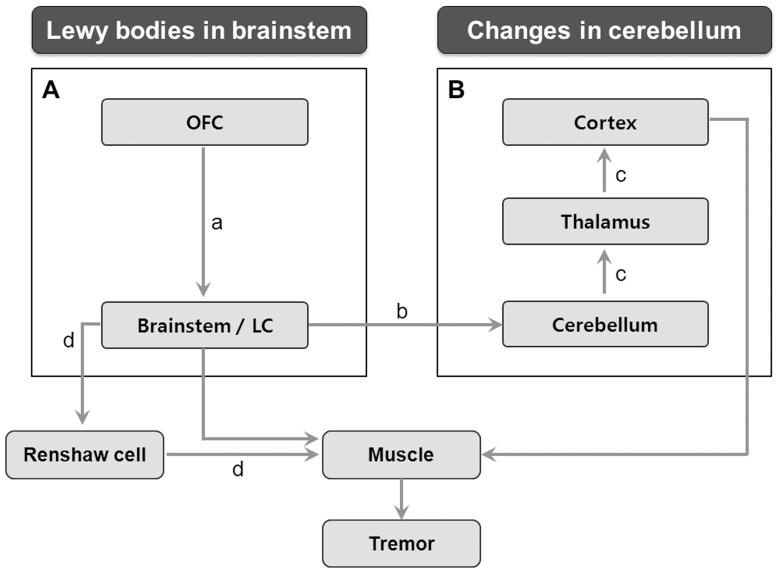
A schematic illustration of the neuroanatomy in the pathophysiology of essential tremor (ET). (A) The locus coeruleus (LC) and its related structure, a major cortical glutamatergic afferent from orbitofrontal cortex (OFC) [a], may play a role in the pathogenesis of ET through the coerulo-cerebellar pathway [b]. (B) The cerebello–thalamo–cortical loop [c] is also an important pathway implicated in ET. These two distinct structures are consistent with heterogeneous neuropathologies (Changes in the brainstem and cerebellum, respectively) demonstrated in previous postmortem studies. Beta blockers seem to act centrally via Renshaw cells [d], and the LC is a strong candidate for mediating the beta-adrenergic effects to this pathway.

In addition to these possible roles of the LC in ET, the LC also projects its noradrenergic innervations to all regions of the brain, including the neocortex, hippocampus, thalamus, subthalamic nucleus, and substantia nigra, and plays roles in several important functions, such as arousal, adaptive gain, and optimal performance [56]. To regulate these functions of the LC, major cortical glutamatergic afferents to LC are known to arise from the orbitofrontal and anterior cingulate cortices [57]. Accordingly, it is possible that this connection between the OFC and LC could have an indirect influence on the pathogenesis of tremor. In the present study, the cortical thinning observed in the OFC of non-responders may have affected the projections to the LC, leading to subsequent alterations of LC functions, which may contribute to the pathogenesis of ET in the non-responder group. More importantly, this alteration in the OFC–LC pathway may lead to changes in a central action of propranolol mediated by the LC, which may explain OFC thinning in non-responders. Although the clinical significance of LC in ET remains uncertain, a few clinicopathological correlation studies indicated that ET patients with LB in the LC tended to have older age of onset, less frequent family history of ET, lower incidence of gait difficulty, and greater likelihood of taking medications for ET than those without LB [7], [8]. Taken together, these results suggest that dysfunction in the LC-related structures may contribute to heterogeneous clinical phenotypes with respect to beta-blocker responsiveness (Figure 3A).

Interestingly, our results of TBSS analysis showed that the responders exhibited more severe disintegration of WM, mainly in the frontal lobe, with relatively preserved cortical thickness in the OFC. This anatomical discrepancy between WM pathology and GM atrophy suggests that the structural changes of WM in the responders would not result from secondary degeneration adjacent to GM abnormalities. Rather, this reflects primary damage in the fibers corresponding with the cerebello–thalamo–cortical loop, which is known to be an important pathway in the pathogenesis of ET. Consistent with these observations, our previous neuroimaging study in different ET patients indicated that patients with ET exhibited reduced FA value of the WM in a similar area in this study compared with healthy controls [14]. Therefore, the present study suggested that responders may have a distinct WM pathology, primarily involving the cerebello–thalamo–cortical loop, with preserved structural integrity around the LC compared with non-responders (Figure 3B).

Previous DTI studies in ET patients have reported inconsistent results in regard to whether the clinical features correlate with WM changes [15], [16], [18]. In our study, correlation analysis indicated that the parameters of tremor severity and disease duration were not significantly correlated with regional cortical thinning or WM alterations. These observations further support the role of propranolol responsiveness as an important factor for ET heterogeneity. In addition, with regard to patterns of clinical parameters in drug responsiveness, there were no significant differences in demographic characteristics or cognitive performance between the two groups in the present study. However, the responder group exhibited more severe WM alterations in the fronto-subcortical circuits that are responsible for cognitive dysfunctions in ET [3], [58]. Thus, a longitudinal study focusing on changes in cognitive performance is needed to confirm this hypothesis.

Some limitations in our study need to be addressed. First, we could not draw a solid conclusion from these imaging data, because this study is not based on pathological data. Second, the relatively small sample size may have limited the detection of group differences. Third, the dosage of 80 mg/day of propranolol may be suboptimal in some cases to declare inefficacy in response. However, previous studies reported that most Asian cases with response showed the efficacy at this dose which may be subtherapeutic in Westerns [59], [60]. Fourth, the idea that the connection between OFC and LC may be involved in the pathogenesis of ET has not been well established yet. Further investigations would be needed to support this hypothesis.

Taken together, the results of the present study suggest that the presence of propranolol responsiveness may be a predictive factor in determining ET subtypes in terms of neuroanatomical heterogeneity. Further large studies with pathological data are required to draw definite conclusions.

## Supporting Information

Table S1 Neuropsychological data in ET patients.
(DOC)
