# Supplementary material for: Neuroanatomical Heterogeneity of Essential Tremor According to Propranolol Response
Source: PLoS One. 2013 Dec 16;8(12):e84054. doi: 10.1371/journal.pone.0084054 (PMC3865260; doi:10.1371/journal.pone.0084054)
Supplement: Table S1 — Neuropsychological data in ET patients. (DOC) [file pone.0084054.s001.doc]

**Table S1. Neuropsychological data in ET patients.**

|  | **Responder**  (n = 18) | **Nonresponder**  (n = 14) | **P** |
| --- | --- | --- | --- |
| **Attention** |  |  |  |
| Digit span (forward) | 6.82 (1.63) | 6.64 (1.22) | 0.533 |
| Digit span (backward) | 3.71 (1.65) | 3.79 (1.19) | 0.897 |
| Digit span total | 10.53 (3.02) | 10.43 (2.17) | 0.776 |
| **Language and related function** |  |  |  |
| K-BNT | 46.47 (9.96) | 46.14 (10.73) | 0.631 |
| Repetition | 14.71 (0.77) | 14.43 (1.16) | 0.250 |
| Calculation | 10.29 (3.20) | 11.07 (1.90) | 0.556 |
| **Visuospatial function** |  |  |  |
| RCFT | 32.50 (5.76) | 34.50 (2.72) | 0.443 |
| **Verbal memory function** |  |  |  |
| Three-word registration | 3.00 (0.00) | 2.93 (0.27) | 0.225 |
| Three-word recall | 2.35 (0.86) | 1.93 (1.00) | 0.200 |
| SVLT |  |  |  |
| Immediate recall | 19.65 (5.24) | 17.29 (6.27) | 0.292 |
| Delayed recall | 6.44 (2.63) | 5.43 (2.10) | 0.314 |
| Recognition | 21.12 (2.40) | 20.29 (1.82) | 0.165 |
| **Visual memory function (RCFT)** |  |  |  |
| Immediate recall | 12.91 (5.59) | 16.54 (5.79) | 0.188 |
| Delayed recall | 12.81 (5.34) | 16.39 (6.24) | 0.163 |
| Recognition | 19.53 (2.13) | 19.92 (1.61) | 0.666 |
| **Frontal executive function** |  |  |  |
| Contrasting program | 19.88 (0.50) | 19.71 (1.07) | 0.534 |
| Go-no-go test | 18.94 (3.04) | 19.57 (1.60) | 0.511 |
| Phonemic generative naming | 23.93 (8.52) | 25.38 (14.41) | 0.822 |
| COWAT (Animal) | 13.92 (4.08) | 15.07 (5.26) | 0.432 |
| COWAT (Supermarket) | 17.59 (6.14) | 17.00 (5.20) | 0.717 |
| Word Stroop test | 110.56 (5.23) | 106.15 (20.49) | 0.314 |
| Color Stroop test | 87.50 (17.24) | 87.92 (28.34) | 0.947 |

The values are expressed as mean (SD). Abbreviations: K-BNT, Korean version of Boston Naming Test; RCFT, Rey Complex Figure Test; SVLT, Seoul Verbal Learning Test; COWAT, Controlled Oral Word Association Test.
